# Dithizone Staining of Intracellular Zinc: An Unexpected and Versatile Counterscreen for Auxotrophic Marker Genes in *Saccharomyces cerevisiae*

**DOI:** 10.1371/journal.pone.0025830

**Published:** 2011-10-05

**Authors:** Daniel S. Yuan

**Affiliations:** Department of Molecular Biology and Genetics, Johns Hopkins University School of Medicine, Baltimore, Maryland, United States of America; Texas A&M University, United States of America

## Abstract

Auxotrophic marker genes such as *URA3*, *LEU2*, and *HIS3* in *Saccharomyces cerevisiae* have long been used to select cells that have been successfully transformed with recombinant DNA. A longstanding challenge in working with these genes is that counterselection procedures are often lacking. This paper describes the unexpected discovery of a simple plate assay that imparts a bright red stain to cells experiencing nutritional stress from the lack of a marker gene. The procedure specifically stains a zinc-rich vesicular compartment analogous to the zinc-rich secretory vesicles found in insulin-secreting pancreatic islet cells and glutamate-secreting neurons. Staining was greatly diminished in *zap1* mutants, which lack a homeostatic activator of zinc uptake, and in *cot1 zrc1* double mutants, which lack the two yeast homologs of mammalian vesicle-specific zinc export proteins. Only one of 93 strains with temperature-sensitive alleles of essential genes exhibited an increase in dithizone staining at its non-permissive temperature, indicating that staining is not simply a sign of growth-arrested or dying cells. Remarkably, the procedure works with most commonly used marker genes, highlights subtle defects, uses no reporter constructs or expensive reagents, requires only a few hours of incubation, yields visually striking results without any instrumentation, and is not toxic to the cells. Many potential applications exist for dithizone staining, both as a versatile counterscreen for auxotrophic marker genes and as a powerful new tool for the genetic analysis of a biomedically important vesicular organelle.

## Introduction

Auxotrophic marker genes such as *URA3*, *LEU2*, or *HIS3* are ubiquitous in yeast genetics, where they are used to select cells that have been successfully transformed with recombinant DNA (reviewed in [1]). By definition, auxotrophic genes are required for growth in the absence of an essential nutrient [2]. For example, mutation of the *URA3* marker gene results in failure to grow in a medium that lacks uracil, as one would expect given that *URA3* encodes a key enzyme in the *de novo* pathway for uracil biosynthesis [3]. Growth of *ura3* mutants in uracil-deficient medium can be restored, however, if the cells have been transformed with DNA containing the *URA3* gene. This exquisite control over growth by the simple presence or absence of a marker gene has been exploited since the earliest attempts to transform yeast cells with recombinant DNA [4].

In themselves, auxotrophic marker genes are not a complete solution for the task of selecting cells that have successfully undergone genetic modification. A complete solution also requires the ability to remove the marker gene, i.e., a counterselection. A widely used counterselection devised for the *URA3* gene involves exposure to 5-fluoroorotic acid [5]. Cells that express *URA3* are inviable, apparently because they accumulate the toxic metabolite 5-fluorouracil [6]. By contrast, *ura3* mutants are unaffected and can be recovered if a small amount of uracil is present in the growth medium. Several useful applications of counterselections involving 5-fluoroorotic acid have been developed, including the replacement of one genetic allele with another using an intermediate state containing *URA3* between the two alleles [7] and the recycling of the *URA3* marker for as many as 20 sequential rounds of transformation [8].

Counterselections for several other auxotrophic marker genes in yeast have also been devised (summarized in [6]). Each involves exposure to a reagent that becomes toxic in the presence of the marker gene. It is important to appreciate that while the underlying strategy is easy to understand, the implementation of a counterselection for any given gene can be difficult. For example, counterselections are lacking for the widely used marker genes *LEU2* and *HIS3*. General-purpose enrichment schemes for auxotrophic yeast strains have also been described. These are based on the increased susceptibility of growing cells to various forms of cell injury due to, for example, membrane permeabilization by Nystatin [9], accumulation of a radioisotope [10], inositol starvation [11], uptake of a DNA-binding peptide [12], or inhibition of cell wall biosynthesis [13]. However, these procedures involve a delicate compromise between adequate cell killing and the ability to recover survivors. Finally, general-purpose counterscreens for auxotrophic yeast strains have also been devised. These procedures stain dead or dying cells with dyes such as phloxine B (Magdala Red) or methylene blue [14]-[17]. An intrinsic weakness of these counterscreens is that the cells that are stained most strongly will also be the least viable.

This paper describes the unexpected discovery of a counterscreen for auxotrophic marker genes based on staining with a cell-permeant zinc-sensing dye, dithizone. Following incubation on selective growth medium, cells lacking a marker gene develop an intense red color upon incubation with dithizone, while cells carrying the required gene are distinctly lighter in color. The stained cells remain viable and easily subcloned, and the procedure works for several auxotrophic marker genes. Remarkably, the zinc-rich vesicles stained by dithizone appear to be the yeast homologs of mammalian zinc-rich secretory vesicles, an intracellular compartment with a key role in the pathogenesis of diabetes and excitotoxic brain injury.

## Materials and Methods

### Construction of a *zap1 zrt1* double heterozygote strain

The homozygous diploid strain YPH274 (*MAT* **a**/**α**, *ura3-52/ura3-52 lys2-801/lys2-801 ade2-101/ade2-101 trp1Δ1/trp1Δ1 his3Δ200/his3Δ200 leu2Δ1/leu2Δ1*; [18]) was first transformed with a *zap1Δ::URA3* deletion construct released from plasmid pDY276 by digestion with *Cla*I and *Not*I [19]. The resulting heterozygous diploid strain was then transformed with a *zrt1::LEU2* disruption construct released from plasmid pZH2 by digestion with *Bam*HI and *Sal*I [20], yielding DYY1141. Each transformation was validated by PCR of genomic DNA from a purified clone, using primer pairs flanking each junction of integration.

### Construction of a *cot1 zrc1* double heterozygote strain

A *zrc1::(lacZ-LEU2-bla*) heterozygous diploid strain (DYY513) was isolated after transposon mutagenesis of YPH274 in the course of a genetic screen for zinc-regulated genes [19]. In this translational *lacZ* fusion, the first 273 codons of *ZRC1* were fused in-frame immediately upstream of *lacZ* coding sequences, and modest induction of *lacZ* activity was observed in low-zinc growth medium (3.0 vs. 0.53 Miller units in low- vs. high-zinc medium, respectively). DYY513 was then transformed with the PCR-amplified *cot1Δ::kanMX4* locus from a *MAT* **a** clone of the Yeast Knockout strain collection (Research Genetics, Huntsville AL, clone ID 1613; [21]), yielding DYY2201. Integration at the *COT1* genomic locus was verified by PCR.

### Assembly of a panel of zinc transport mutants

Haploid mutant strains were obtained by sporulation and tetrad dissection of the double heterozygous mutant strains DYY1141 and DYY2201 described above. The *zap1 zrt1* series of strains shown were *MAT* **α**; the *cot1 zrc1* strains were *MAT* **a**. All eight strains were otherwise isogenic.

### Assembly of a Haploid Progeny Collection

The parent strain of this collection, DYY2017, was derived from BY4743, the progenitor of the heterozygous diploid Yeast Knockout strain collection [21], [22]. DYY2017 contains six heterozygous selectable marker genes: *LYS2* and *MET15* from BY4743; *can1Δ::LEU2-MFA1pr-HIS3*, a *MAT* **a**-specific selectable marker [23] (the genetically unlinked *MAT* and *CAN1* loci are scored with the *HIS3* and *LEU2* selectable markers, respectively); and two selectable markers integrated as deletion constructs at phenotypically silent loci, *hoΔ::kanMX4* (from the Yeast Knockout strain collection) and *ura3Δ0::loxP-URA3MX* [24]. Integration close to the *ura3* locus was demonstrated by the lack of Ura^-^ meiotic progeny in 12 tetrads obtained after mating a *URA3MX* haploid strain with a *URA3* mating-type tester strain (see below).

Haploid progeny representing all 64 combinations of the six selectable markers were isolated from colonies obtained by sporulation of DYY2017 and tetrad dissection. Mating types of the two Leu^+^ Ura^+^ (G418^S^ or G418^R^) His^+^ Lys^+^ Met^+^ progeny strains could not be determined by mating with a *MAT* **α** *thr4* tester strain, because the unmated strains grew well on the Minimal defined medium used to select mated cells. They were assigned *MAT* **a** mating type based on their expression of the *MAT* **a**-specific genetic marker, and on their haploid rather than diploid cellular morphology.

The strains were ordered by phenotype and assembled in an 8×8 array, with no-growth and growth phenotypes for each marker gene alternating in blocks running down or across the array. Leu^-^ and Leu^+^ strains are in blocks of four rows; Ura^-^ and Ura^+^, two rows; G418^S^ and G418^R^, single rows; His^-^ and His^+^, four columns; Lys^-^ and Lys^+^, two columns; and Met^-^ and Met^+^, single columns. Also included in the collection are four colonies of DYY2017 (above the array) and two colonies each of the (non-congenic) mating-type tester strains F243 and F244 (*MAT* **a** *thr4* and *MAT* **α** *thr4*, respectively; originally from G. Fabian via Alan Hinnebusch; [25]) (right of the array). The collection should be maintained on YPD medium (see below) with extra histidine added (0.3 mM) to avoid inadvertent selection for His^+^ cells in the diploid strain, which emerge at a high frequency.

### Rich media for yeast growth

Rich medium consisted of Bacto Peptone 2% w/v, Bacto Yeast Extract 1% w/v, and glucose 2% w/v (YPD), plus tryptophan 1.5 mM. “Supplemented rich medium” contained additional adenine hemisulfate 80 mM, to suppress the accumulation of red pigments in *ade2* strains, and methionine 0.6 mM, to reduce background dithizone staining in *met15* strains.

### Defined media for yeast growth

Synthetic defined medium was as defined in Table 3 of [26], except that *myo*-inositol and *para*-aminobenzoic acid were absent from the Yeast Nitrogen Base mixture, and that nutrient dropout mixtures included Thr but not Cys. Specifically, nutrient dropout mixtures consisted of a basal mixture of amino acids (Ala, Arg, Asp, Asn, potassium glutamate, Gln, Gly, Ile, Phe, Pro, Ser, Thr, Tyr, and Val) combined with any nutrients needed for missing auxotrophic markers (uracil, Leu, His, Trp, Lys, Met, and/or adenine hemisulfate). “Minimal defined medium” contained no nutrient dropout mixture. “Supplemented defined medium” contained additional adenine and methionine, as in supplemented rich medium, plus succinic acid 10 mM and potassium bicarbonate 15 mM to buffer the pH at 5.0.

All solid media had the same composition as the liquid media described above except that they included 2% w/v Bacto Agar.

### Dithizone stock solutions

The main formulation of dithizone in this paper was prepared by adding 200 µl ethanol and 40 µl concentrated ammonium hydroxide to 20 mg dithizone (Sigma D5130) (following [27] but omitting 4-methylpyridine). The clear dark-red solution was then diluted with 800 µl 10% Triton X-100 in water [28] and stored under inert gas at −20°. The Triton detergent greatly retarded the formation of precipitates, although a brown sediment eventually appeared after several cycles of freezing and thawing even if the solutions were kept at 4° or below. Tween-80 could be used in place of Triton X-100, but yeast colonies stained with dithizone-Triton exhibited a wider range of colors that appeared to be informative.

**Figure 1:**
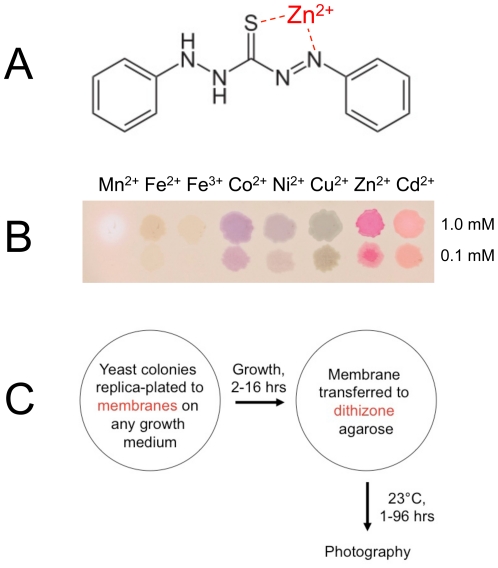
Overview of the dithizone staining procedure. (**A**) Chemical structure of dithizone. The zinc ion was placed at the crystallographically determined ligand binding site for zinc [62]. (**B**) Colored dithizone complexes formed with some transition metal ions. Droplets of 1 mM or 0.1 mM metal sulfate solutions (except chlorides for Co^2+^ and Ni^2+^) were applied to a nitrocellulose membrane on a dithizone–DMSO-agarose plate and photographed after one hour at 23°. (**C**) Schematic of a plate assay for staining yeast colonies.

**Figure 2:**
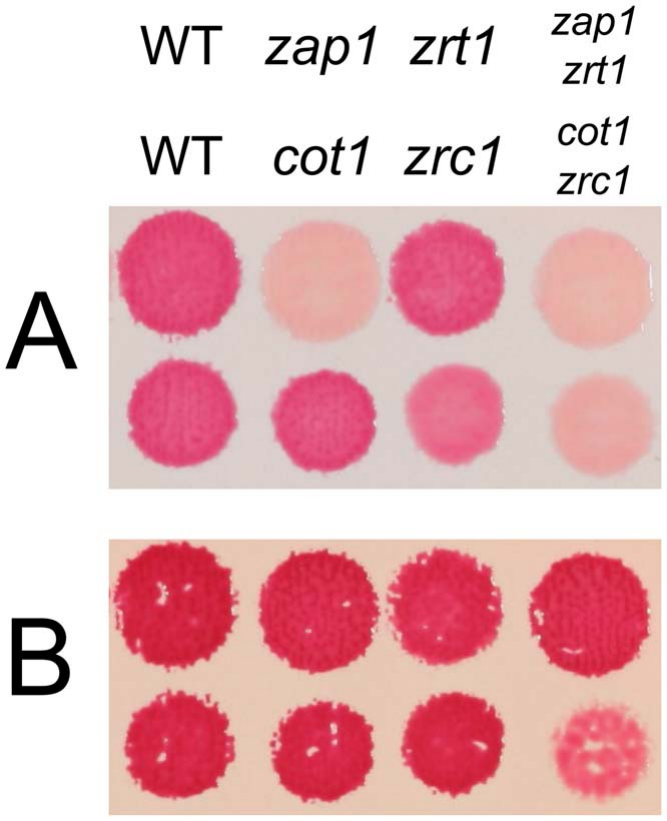
Dithizone staining defects in zinc transport mutants. (**A**) Staining of patches of yeast cells grown on a nitrocellulose membrane on supplemented rich medium (see Materials and Methods) after transfer to dithizone-DMSO-agarose plates. (**B**) Same as in part A but with ZnSO_4_ 300 µM also added.

**Figure 3:**
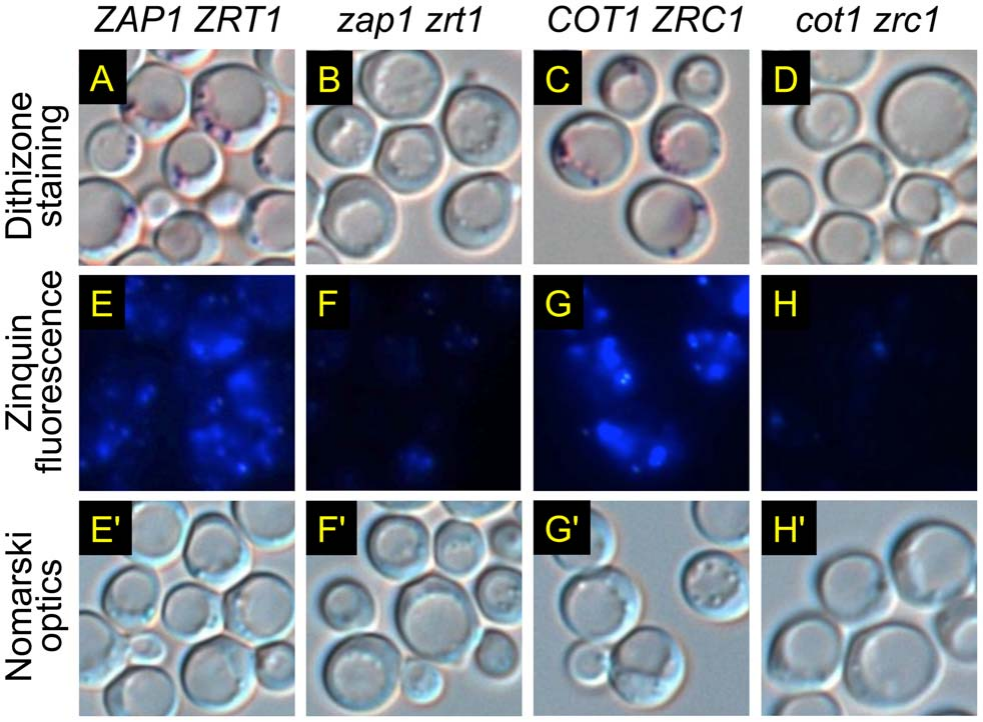
Effects of zinc transport mutations on dithizone or Zinquin staining of a vesicular compartment. The indicated strains were grown overnight to saturation in a supplemented defined medium (see Materials and Methods) containing 30 µM ZnSO_4_. Zinc-rich vesicles were visualized by two different staining methods as indicated. Cells in Panels E-H correspond to those in Panels E'-H'.

An alternative formulation of dithizone, used in Figures 1, 2, 3, and S1A of this paper, was prepared by dispersing 20 mg dithizone with 1 ml DMSO (dimethylsulfoxide), following [29], but then adding 100 µl of 1 M Tris base (*tris*(hydroxymethyl)-aminomethane) instead of a phosphate buffer. Incremental additions of Tris base revealed an abrupt transition from a dark green suspension to a dark red solution, suggesting the formation of an equimolar dithizone/Tris complex. Comparable amounts of 1 M K_2_CO_3_ or K_2_HPO_4_ failed to reproduce the effects of Tris base, indicating that alkalinization is not the principal means by which Tris base appears to stabilize dithizone in solution. Stock solutions were stored under inert gas at −20° and were easily thawed in an ice bath despite their high DMSO content. Solutions could be frozen and thawed repeatedly and could be stored for three weeks at 23° with no apparent change.

### Dithizone-agarose plates

A base solution were prepared by adding Tris base 50 mM and sodium azide 1 mM to a 1% w/v water solution of melted agarose (GenePure LE, Fisher Scientific). Special grades of agarose with low metal content were unnecessary, and other agarose preparations for molecular biology are probably suitable. The Tris base and sodium azide were included to neutralize and block cellular metabolic activity, which would otherwise acidify the agarose and cause the dithizone to precipitate. To facilitate cooling, the agarose solution was microwaved to boiling in one-fourth the volume, diluted to full volume, and then briefly swirled in tap water until the flask was only mildly warm to touch. Tris base, sodium azide, and dithizone stock solution (1/100 volume for dithizone in DMSO or 1/500 volume for dithizone in Triton solution) were then individually added and swirled rapidly into the agarose solution. Plates were filled without delay and were inverted and stored in the dark at 4° immediately after hardening of the agarose. Immediately before use, the plates were thoroughly drained to remove all free liquid. Stored plates gradually lost their bright orange color and became ineffective for staining, especially if the dithizone solution had been too warm. Plates containing Triton X-100 had a shelf life of about three weeks; those with DMSO, about three days.

### Dithizone plate assay

Yeast colonies were replica-plated to a membrane on a plate of growth medium that had been air-dried to remove all free fluid. Air bubbles trapped under the membrane were best removed before replica-plating by peeling up one side of the membrane and carefully laying it down again. After sufficient growth to distinguish auxotrophic from prototrophic colonies (usually 16 hours at 30°), the membrane was slowly lifted off the plate using forceps. To minimize wrinkling and disruption of the colonies, this was done with the plate tilted at an 80-degree angle and the membrane kept as flat as possible. The membrane was placed onto a dithizone plate, reversing the steps used in lifting the membrane and carefully freeing any trapped air bubbles. The plate was incubated face up in the dark at 23° until positive and negative controls could be readily distinguished. Staining was complete in about one hour for nitrocellulose membranes and six hours for nylon membranes (see below). Stained plates could be left at 23° for 1–2 days or stored at 4° for several days with only minor changes in appearance.

Yeast colonies were most reliably assessed by dithizone staining if they were large and flat. Such colonies were best prepared by spotting 3 µl of a cell suspension (1-10 OD_600_/ml) onto growth medium, waiting for the liquid to be absorbed, and incubating overnight. Alternatively, colonies were replica-plated first to a fresh plate of growth medium, to “debulk” the excess colony biomass on the velvet (“replica-cleaning” [30]), before printing them to membranes.

A variety of membranes developed for colony hybridization applications were found to be suitable for dithizone staining, including nylon (Roche, catalog number 1 699 075; Schleicher and Schuell, catalog numbers 77416 or 77408) and supported nitrocellulose (Schleicher and Schuell, catalog number 68290), with pore sizes of either 0.45 µm or 0.2 µm. Nylon membranes were more durable and easier to handle than nitrocellulose membranes, but they retained a pale orange background stain, took several times longer to stain, and were also generally more expensive. Membranes to fit OmniTrays (rectangular plates in SBS “96-well” format; Nalge Nunc, catalog number 242811) were cut from 30 cm-wide sheets to 114×75 mm^2^ rectangles using a rotary paper trimmer; strict sterile technique was not necessary. Dithizone staining was also feasible with filter papers, e.g., Whatman 1, 3MM, or 542, but the colonies were harder to see against the translucent wet paper.

### Dithizone and Zinquin staining of cells for microscopy

For each coverslip to be viewed by microscopy, 1 OD_600_ of cells was pelleted from liquid culture (23°, 1000×*g* 1 min). The cells were immediately resuspended in 1 ml of a solution (“buffer”) of Tris base 50 mM and sodium azide 1 mM, pelleted, and rewashed as needed to remove traces of the culture medium. Cells to be stained were suspended in 50 µl buffer, diluted with a mixture of 50 µl buffer and 1 µl dithizone stock solution in DMSO, mixed gently, and incubated at 23° for 60 min with occasional mixing. The cells were then washed with buffer, resuspended in 3 pellet volumes of residual supernatant, and prepared for microscopy. Zinquin-stained cells were prepared the same way except that the dithizone stock solution was replaced with a premix of 1 µl DMSO and 1 µl of Zinquin ethyl ester (Luminis Pty Ltd., Adelaide, South Australia) delivered as a 5 mg/ml solution in absolute ethanol. Zinquin stock solutions were stored in sealed tubes in the dark at 23° for more than 12 years without an obvious deterioration in staining performance.

## Results

### Development of a methodology for dithizone staining

Dithizone (Figure 1A) has been used for several decades as an analytical reagent for zinc. As a solution in carbon tetrachloride [31] or chloroform [32], however, its usual formulation is incompatible with biological samples. The challenge of solubilizing and stabilizing dithizone in aqueous buffers suitable for staining cells *in vivo* was solved by using a formulation of dithizone in ethanol, ammonium hydroxide, and the detergent Triton X-100 (see Materials and Methods). Most of the studies in this paper were carried out using this formulation in a plate assay format.

It later emerged that Triton solutions of dithizone were incompatible with yeast microscopy. Cells directly suspended in staining buffers exhibited a gradual loss of refractility of the vacuole in solutions containing 0.1% w/v Triton, consistent with the gradual permeabilization of intracellular organelles by the detergent. This finding led to the development of a second formulation of dithizone that would be suitable for staining cell suspensions. This formulation employed DMSO and Tris base (see Materials and Methods) and is also used in this paper.

To characterize the staining properties of dithizone in a format conducive to yeast genetics, droplets of various metal ions were applied to agarose gels containing dithizone (see Materials and Methods). The resulting metal-dithizonate complexes produced a veritable rainbow of colors (Figure 1B). Importantly, zinc was the only biologically relevant metal ion that exhibited persistent red or magenta staining. Manganous and ferrous ions stained pink or purple initially but these colors faded quickly, ending up as a negative stain or brown precipitate, respectively.

Dithizone-agarose gels were then incorporated into a simple colony-lift assay for staining a plate of yeast colonies *in situ* (Figure 1C). See the Materials and Methods for details of this assay.

### Genetic analysis of cellular dithizone staining

To characterize dithizone staining of yeast cells in genetic terms, mutants with defects in zinc uptake (*zap1*, *zrt1*) or zinc export (*cot1*, *zrc1*) were assembled into a panel for testing on a variety of growth media.

*ZAP1* encodes a transcription factor in *S. cerevisiae* that is responsible for zinc homeostasis [33]. *ZRT1* encodes a high-affinity zinc uptake transporter [20] that is a homolog of Zip1 through Zip14, comprising the SLC39 family of human zinc uptake transporters [34]. In zinc-deficient cells, Zap1p activates the transcription of several genes involved in zinc uptake, including *ZAP1* itself and *ZRT1*, and mutations in *ZAP1* and *ZRT1* each result in a zinc-deficient state that responds to zinc supplementation [35]. One would therefore expect the *zap1 zrt1* double mutant to be at least as zinc-deficient as the *zap1* single mutant.

*COT1* and *ZRC1* encode putative zinc export proteins in yeast, each of which contains multiple predicted membrane-spanning domains. *COT1* and *ZRC1* were each identified by their ability to increase zinc resistance when overexpressed [36], [37]. When expressed from their genomic loci as carboxyl-terminal GFP fusion proteins, Cot1p and Zrc1p are localized to the vacuolar membrane [38]. One would therefore expect the *cot1 zrc1* double mutant to exhibit an exaggerated sensitivity to zinc. Cot1p and Zrc1p are the two *S. cerevisiae* homologs of ZnT1 through ZnT9, comprising the SLC30 family of human transport proteins that export zinc out of the cytosol, either across the plasma membrane or into a vesicular compartment [34]. For example, zinc export into the insulin-containing secretory vesicles of pancreatic islet beta cells is mediated by ZnT8, a member of this family [39], while the same process in many glutamate-secreting neurons of the central nervous system is mediated by ZnT3, another member of this family [40]. One might therefore expect the *cot1 zrc1* double mutant to be impaired in vesicular zinc export as well.

After growth on a rich medium, wild-type colonies stained with dithizone developed a deep red color against an off-white background. Both *zap1 zrt1* and *cot1 zrc1* double-mutant colonies remained beige (Figure 2A). The *zap1 zrt1* double mutant and *zap1* single mutant showed a similar decrease in staining, consistent with the strong transcriptional activation of *ZRT1* by Zap1p. By contrast, the *cot1* and *zrc1* single mutants resembled wild-type cells in both growth and dithizone staining and were easily distinguished from the *cot1 zrc1* double mutant. These findings implicated Cot1p and Zrc1p as transport proteins that together are chiefly responsible for the color obtained during dithizone staining.

Modest zinc supplementation (300 µM) (Figure 2B) intensified wild-type staining to a dark red color. Of note, *zap1 zrt1* cells were now stained as intensely as wild-type cells. With *cot1 zrc1* cells, however, zinc supplementation not only failed to restore dithizone staining to wild-type levels but also inhibited growth dramatically. These findings confirmed that Cot1p and Zrc1p together also have an essential function in mediating cellular zinc resistance.

Light microscopy revealed intensely colored granules in the stained colonies of wild-type cells (Figures 3A,C), but not of double-mutant cells (Figures 3B,D). Similar staining patterns were observed (Figures 3E-H) when other aliquots of the same cells were incubated with Zinquin, a zinc-specific fluorescent dye. Zinquin has been used to visualize a zinc-rich vesicular compartment in yeast [29], [34], [41], and it brilliantly stains zinc-rich secretory vesicles in a wide range of mammalian cell types [42], [43]. The clear differences in staining between *cot1 zrc1* mutants and wild-type cells were surprising given the lack of such differences in previous studies [29], [34]. These discrepancies could be due to differences in genetic background or staining methodology and have not been resolved. Nevertheless, the findings here provided genetic evidence that the red-purple color visualized by dithizone staining is specific for zinc. Interestingly, the Zinquin-stained vesicles visualized here appeared to co-localize with tiny refractile bodies seen by Nomarski optics, within and around the vacuole (Figures 3E'-H').

### Discovery of a dithizone staining phenotype of auxotrophic cells

A potential concern in any study of gene disruption mutants is that the phenotypes of a gene disruption may be confounded by phenotypes of the marker gene used to construct the disruption [2]. Indeed, three different marker genes were used among the four gene disruptions shown in Figure 2. To ascertain whether marker genotypes influence dithizone staining, haploid progeny representing all 64 combinations of genotypes from a six-way heterozygous diploid strain were isolated by tetrad dissection (see Materials and Methods). The resulting “Haploid Progeny Collection” was replica-plated to a variety of marker-selective, synthetic defined media (lacking uracil, leucine, histidine, tryptophan, lysine, methionine, or adenine) and assessed for growth and dithizone staining.

**Figure 4:**
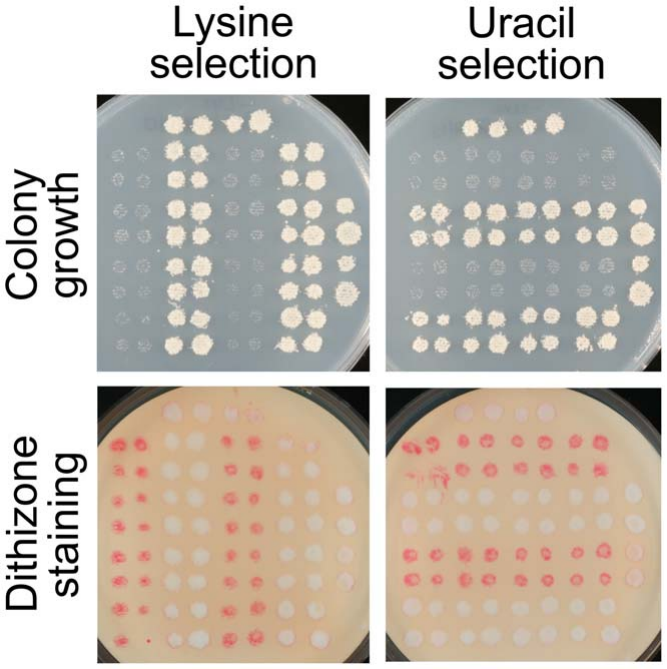
Dithizone staining of auxotrophic strains. The Haploid Progeny Collection (see text) was replica-plated to the indicated selective media. Growth and staining were assessed with different series of replicas. Auxotrophic strains, i.e., strains that grew poorly on selective medium, were bright red after staining on dithizone-Triton-agarose plates. Images are unenhanced.

The unexpected and remarkable finding from this experiment was that the cells of auxotrophic strains on each growth medium were stained bright red. Representative plates are shown in Figure 4. The red “auxotrophic staining” contrasted vividly with the nearly white colonies of prototrophic strains. The difference in the level of wild-type staining (comparing Figures 3 and 4) is attributed to the much lower zinc content of defined medium compared with rich medium, although accurate estimates are not available due to the substantial quantities of metal ions present in agar and other bulk components of growth media. Curiously, auxotrophic staining was also observed in strains that were sensitive to 5-fluoroorotic acid, due to counterselection of the *URA3* marker gene. Auxotrophic staining was not simply the result of the scant biomass transferred by replica plating, because no such staining was observed when the tops of well-grown colonies were scraped away.

**Figure 5:**
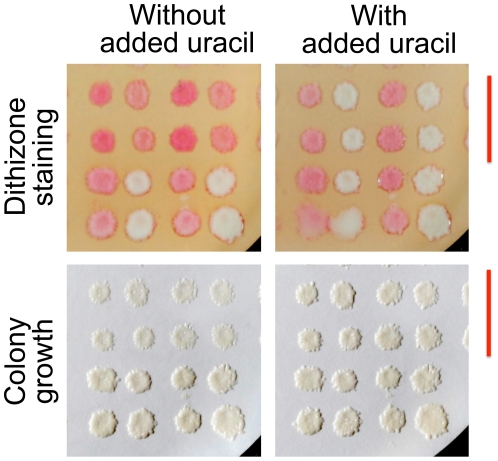
Discovery and correction of partial uracil deficiency in a methionine-poor growth medium. The Haploid Progeny Collection used in Figure 4 was replica-plated onto a nylon membrane on the medium, without or with added uracil. After overnight growth, the colonies were photographed (lower Panels), stained on dithizone-Triton-agarose plates, and photographed again (upper Panels). For clarity, only the lower right quadrant of the collection is shown (*LEU2 HIS3* cells). Image contrast was increased using the same adjustments for the paired uracil conditions. Red bars denote rows of uracil auxotrophs; columns alternate between methionine auxotrophs and prototrophs.

The predictive power of dithizone staining was illustrated by its ability to detect unsuspected and subtle nutrient deficiencies in growth media. One of the growth media tested had been formulated to enhance mutant phenotypes of the *MET15* (now *MET17*) marker gene [44]. Cells lacking a functional *MET15* gene emit hydrogen sulfide due to a block in methionine biosynthesis, and plate assays for *met15* mutants based on the black color of metal sulfides were optimized to exacerbate the block by driving methionine biosynthesis. As expected, the *met15* strains (methionine auxotrophs) exhibited enhanced dithizone staining, consistent with mild methionine deprivation (Figure 5, first and third columns in each panel). Surprisingly, however, dithizone staining was also enhanced in the *ura3* strains (uracil auxotrophs), suggesting that the medium contained suboptimal amounts of uracil (Figure 5, rows marked by a red bar). Colonies photographed before staining exhibited only subtle changes that easily could have been missed on a plate full of colonies. Uracil supplementation abolished both the growth defect and the auxotrophic staining that had occurred with the *ura3* strains (Figure 5).

### Evidence for mechanistic specificity in auxotrophic dithizone staining

Given the wide variety of selective growth media capable of eliciting auxotrophic staining, it was natural to ask if auxotrophic staining was simply a sign of growth-arrested or dying cells. To test this hypothesis, cells were grown to saturation on a low-glucose medium and stained with dithizone. Dithizone staining was decreased, not increased (Figure S1A). Similar results were obtained with a spontaneously respiratory-deficient mutant that could not utilize ethanol as its principal carbon source. Dithizone staining in this mutant was also decreased (Figure S1B). Thus, auxotrophic staining could not be elicited by glucose restriction.

**Figure 6:**
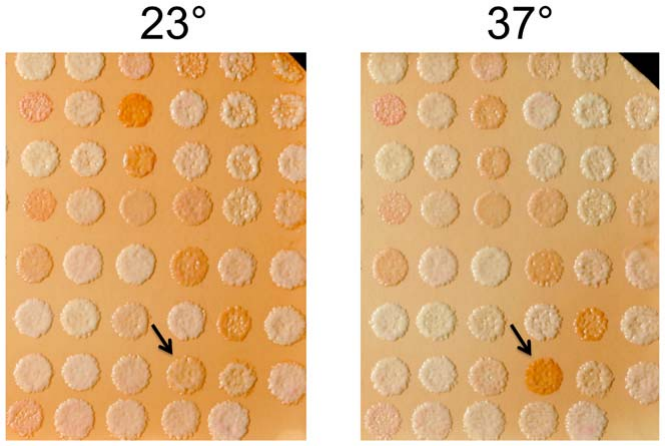
Dithizone staining of a panel of temperature-sensitive mutants of essential genes. Patches of each strain were replica-plated to duplicate nylon membranes on a supplemented defined medium (see Materials and Methods) and grown at 23° for 18 hours. One membrane was then transferred to a plate of fresh medium and warmed to 37° for four hours. The other membrane was treated identically except it was kept at 23°. Membranes were then transferred to dithizone-Triton-agarose plates at 23° and photographed after three days. For clarity, only the right half of each membrane is shown. Contrast was enhanced for both images using the same transformation. Arrows point to *guk1*, the only strain that exhibited increased dithizone staining at the non-permissive temperature.

The relationship between growth arrest and dithizone staining was also examined using a strain collection containing temperature-sensitive alleles of essential genes. Virtually all of these strains undergo growth arrest at a non-permissive temperature of 37° [45]. A representative plate of 93 strains was replicated to membranes on duplicate plates with no missing nutrients and grown overnight at the permissive temperature (23°). One plate was then transferred to a 37° forced-air incubator while the other was left at 23°. After four hours, dithizone staining revealed that only one of the strains had developed an increase in staining at the non-permissive temperature (Figure 6). The gene mutated in the affected strain, *GUK1*, encodes guanylate kinase, an essential step in the purine salvage pathway. Thus, starvation for purines in the *guk1* strain could account for the observed increase in dithizone staining. The only other strain on the plate that might also be auxotrophic for a specific nutrient was *rib2*, with a block in riboflavin biosynthesis, but it did not exhibit increased dithizone staining at its non-permissive temperature. These inconsistencies notwithstanding, the overall paucity of mutants exhibiting increased dithizone staining at their non-permissive temperature established that auxotrophic staining is not simply a sign of growth-arrested or dying cells.

## Discussion

This paper introduces dithizone staining as a new assay of nutritional stress in auxotrophic yeast strains. The assay imparts a bright red color to cells that have become nutrient-deficient due to their inability to produce the nutrient endogenously or to acquire it in adequate amounts from the growth medium. The color appears to represent a previously unsuspected state of zinc mobilization that is actively induced in response to nutrient deficiency. The mechanism of induction is unknown but presumably requires signaling between one or more of the nutrient-sensing pathways in yeast [46] and the as yet uncharacterized mechanisms that control the biogenesis and trafficking of zinc-rich vesicles within the yeast secretory pathway.

As a counterscreen, dithizone staining has the important limitation that it does not enrich the population for auxotrophic cells. Because the auxotrophs must first be stained as colonies before they can be isolated, the feasibility of dithizone staining for a particular task will be inversely proportional to the number of colonies that must be screened. Practical constraints arise from the simple fact that a plate of growth medium can hold only a few thousand colonies before they become indistinguishable. Thus, cells that have spontaneously lost a centromeric plasmid may be easy to isolate (requiring ∼10^3^ colonies), but mutants from a saturated screen of the Yeast Knockout strain collection may be more challenging to find (∼10^5^ colonies), while integrative transformants, informative point mutants, and other rare clones probably cannot be isolated by dithizone screening alone (∼10^7^ colonies). Nevertheless, dithizone staining has three strengths that should not be overlooked.

First, the success of a genetic screen based on dithizone staining does not depend on toxic metabolites to inhibit the growth of prototrophic cells. This fact bypasses potential issues associated with a growth inhibition strategy, such as suboptimal dosing, drug resistance mechanisms, or the emergence of suppressor mutations. Indeed, dithizone staining is associated with little if any toxicity. Dithizone-stained yeast cells can be subcultured directly from the membrane with excellent viability and vigor (Figure S2). Significantly, dithizone staining already has an established record of safety in clinical settings as a technique for identifying pancreatic islet tissue prior to organ transplantation [47].

Second, dithizone staining is remarkably versatile. A single procedure works for most auxotrophic marker genes, and the procedure appears to rely only on the function of endogenous mechanisms for zinc transport, mechanisms also found in the distantly related yeast *Schizosaccharomyces pombe* [48] as well as in higher eukaryotic organisms [34]. Gene-specific reagents are unnecessary, and no special genetic mutations or reporter-gene constructs need to be introduced into the cells being studied. The only commonly used auxotrophic marker gene that is currently known to be incompatible with dithizone staining is *ADE2*. The red pigment that accumulates in *ade2* cells appears to interfere with the development of red dithizone staining, not only because it has a similar hue but also because pigmented cells exhibit weak dithizone staining compared with cells grown with adequate adenine.. This is not a serious drawback, however, because the red pigment in these mutants is itself a counterscreen for certain adenine auxotrophs and is already a familiar tool in yeast genetics [49], [50], [51].

Third, dithizone staining is capable of discerning graded responses to nutritional stress that may not be obvious from visual assessments of growth alone. Subtle distinctions in color hue or saturation are easily and reproducibly detected with the unaided eye, and the plate assay format lends itself to comprehensive experiments with numerous replicates and controls, experiments that would be cumbersome to perform with liquid cultures. Through its ability to detect nutritional stress in its early stages, as illustrated in Figure 5, dithizone staining can be thought of as a “red flag” for mismatches between the nutritional needs of the organism and the nutritional resources provided to it. It is not difficult to imagine practical applications for dithizone staining, such as quality control procedures for a media kitchen or selection strategies for the incremental refinement of engineered yeast strains.

An important question for future research is whether the scope of application for auxotrophic staining excludes certain classes of nutrients, e.g., bulk nutrients such as phosphate or trace nutrients such as vitamins. This idea arises from recent revelations that sharp distinctions can be made between starvation for bulk nutrients and starvation for auxotrophic nutrients [e.g., 52,53,54]. A second question is whether it is possible for multiple concurrent auxotrophies to interfere with one another, rather than merely combine additively (as illustrated by the methionine and uracil auxotrophies in Figure 5). A third question concerns whether the intensity of dithizone staining reflects the *abruptness* of change in nutrient availability rather than the current level of nutrient availability, i.e., whether dithizone staining should be regarded as a dynamical system with viscoelastic properties rather than as a static readout of a genetic trait. Early experiments appear to answer each of these questions in the affirmative. For example, glucose restriction does not induce auxotrophic staining (Figure S1).

In addition to its potential applications as a technical tool in yeast genetics, dithizone staining opens a new window on the biology of zinc-rich vesicles. This is a counterintuitive notion because detailed studies of zinc-rich vesicles (sometimes termed “zincosomes,” after [55]) have long since abandoned dithizone as a zinc sensor in favor of fluorescent reagents [56], [57]. The genetic evidence in this paper indicates, however, that dithizone staining is a specific method for detecting zinc-rich vesicles *in vivo*, at least in yeast cells grown in conventional growth media. Moreover, unlike fluorescent reagents, dithizone is cheap, slow to fade, and easily visualized by the unaided eye. These practical advantages have spawned genetic screens that are currently underway.

Zinc-rich vesicles have deep connections to clinical medicine. Major examples include the pathogenesis of diabetes, both type 1 and type 2 (e.g., [58], [59]), and the mechanisms of brain injury after hypoxia and stroke (e.g., [60], [61]). Many fundamental questions remain to be answered, including how zinc is concentrated within zinc-rich vesicles and released to the cytosol, and how zinc-rich vesicles interact with the secretory pathway in both physiological and disease states. Through the systematic analysis of yeast mutants, dithizone staining may help to illuminate the physiology and cell biology of this important organelle.

## Supporting Information

Figure S1 **Lack of increased dithizone staining in glucose-restricted cells. **(**A**) Patches of yeast cells were replica-plated to supplemented rich media containing either standard (2% w/v) or low (0.2% w/v) concentrations of glucose and prepared for dithizone staining. The top Panel is the same as Figure 2A; the bottom Panel was processed concurrently. (**B**) Patches of a parental strain with the *ade2* genetic marker and an isogenic spontaneously-arising petite mutant (isolated by its white rather than pink colony color) were replica-plated to nylon membranes on supplemented rich medium containing either a standard (glucose 2% w/v) or nonfermentable (ethanol 3% v/v) carbon source. After growth at 30° for one day, the cells were stained on dithizone-Triton-agarose plates.
(TIF)

Figure S2 **Lack of toxicity from dithizone staining.** Patches of wild-type or mutant yeast cells were replica-plated to duplicate membranes and prepared for dithizone staining as in Figure 2A. One membrane was incubated on an agarose plate containing dithizone in DMSO as in Figure 2A. The other membrane was incubated on a duplicate plate prepared identically except with DMSO alone. The indicated patches were harvested from the membranes, subjected to serial ten-fold dilutions in a buffer consisting of Tween-80 0.1%, BHT 1 ppm, glucose 5% w/v, and sodium citrate 50 mM pH 6.5, and spotted on a conventional YPD agar plate. The last two spots in each row of dilutions were expected to have about 3 or 0.3 colonies, respectively, based on the optical density of each harvested cell suspension and assuming 100% viability. The pink color is typical of the *ade2* genetic marker present in these strains.
(TIF)
