# Supplementary figures and images for: Dithizone Staining of Intracellular Zinc: An Unexpected and Versatile Counterscreen for Auxotrophic Marker Genes in Saccharomyces cerevisiae
Source: PLoS One. 2011 Oct 5;6(10):e25830. doi: 10.1371/journal.pone.0025830 (PMC3187812; doi:10.1371/journal.pone.0025830)

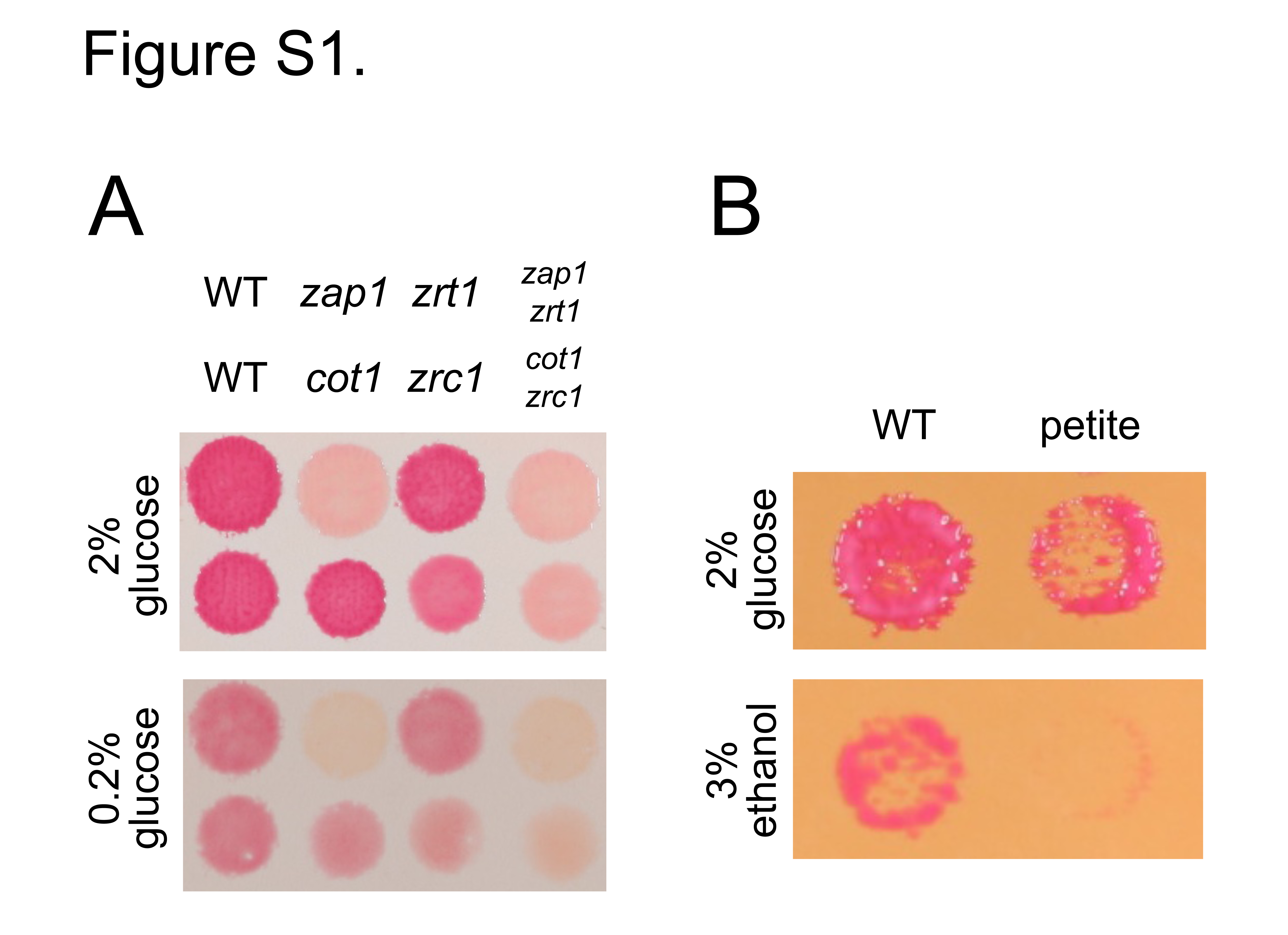

Supplement: Figure S1 — Lack of increased dithizone staining in glucose-restricted cells. (A) Patches of yeast cells were replica-plated to supplemented rich media containing either standard (2% w/v) or low (0.2% w/v) concentrations of glucose and prepared for dithizone staining. The top Panel is the same as Figure 2A; the bottom Panel was processed concurrently. (B) Patches of a parental strain with the ade2 genetic marker and an isogenic spontaneously-arising petite mutant (isolated by its white rather than pink colony color) were replica-plated to nylon membranes on supplemented rich medium containing either a standard (glucose 2% w/v) or nonfermentable (ethanol 3% v/v) carbon source. After growth at 30° for one day, the cells were stained on dithizone-Triton-agarose plates. (TIF) [file pone.0025830.s001.tif]

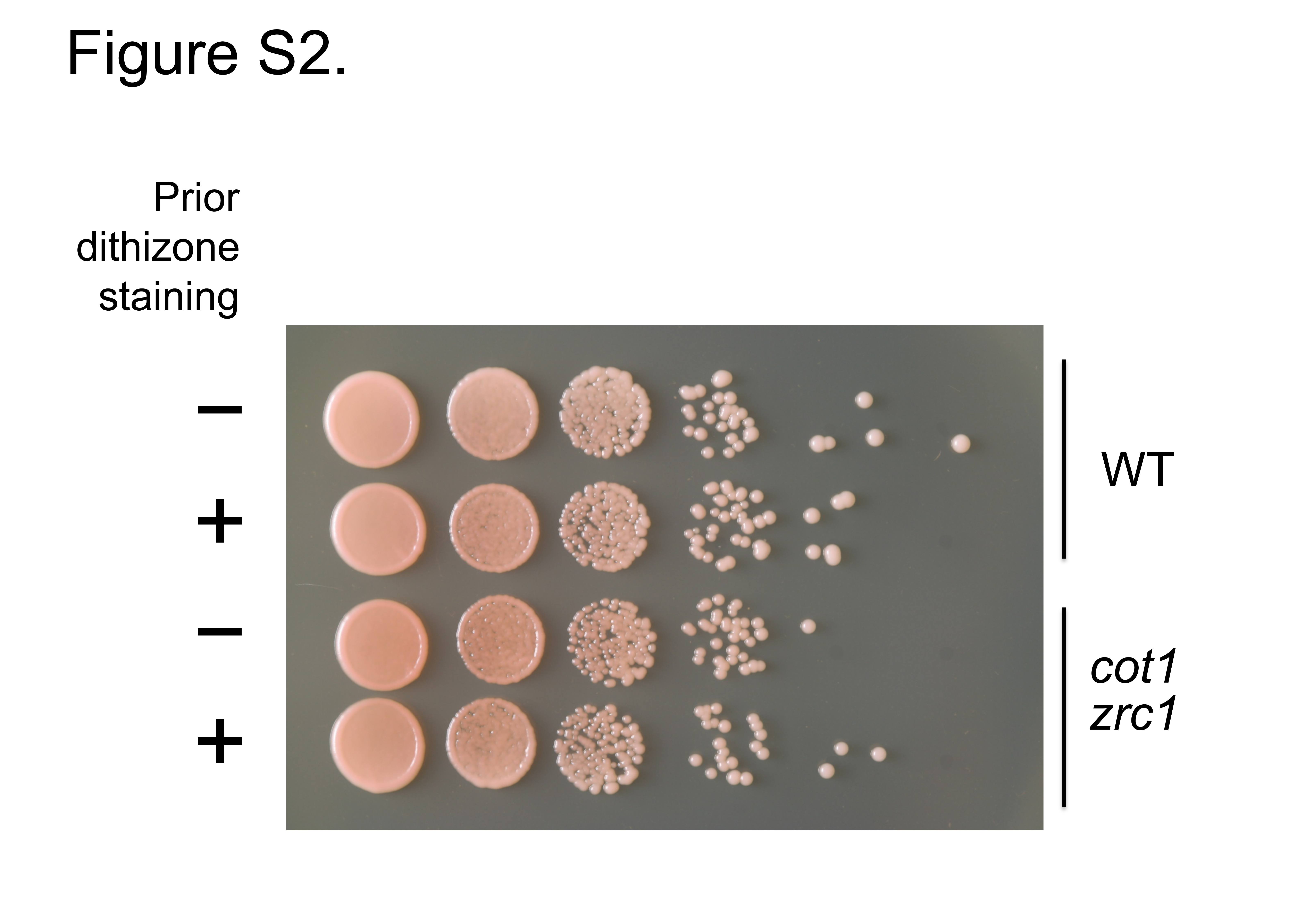

Supplement: Figure S2 — Lack of toxicity from dithizone staining. Patches of wild-type or mutant yeast cells were replica-plated to duplicate membranes and prepared for dithizone staining as in Figure 2A. One membrane was incubated on an agarose plate containing dithizone in DMSO as in Figure 2A. The other membrane was incubated on a duplicate plate prepared identically except with DMSO alone. The indicated patches were harvested from the membranes, subjected to serial ten-fold dilutions in a buffer consisting of Tween-80 0.1%, BHT 1 ppm, glucose 5% w/v, and sodium citrate 50 mM pH 6.5, and spotted on a conventional YPD agar plate. The last two spots in each row of dilutions were expected to have about 3 or 0.3 colonies, respectively, based on the optical density of each harvested cell suspension and assuming 100% viability. The pink color is typical of the ade2 genetic marker present in these strains. (TIF) [file pone.0025830.s002.tif]
